# Supplementary material for: Does cotrimoxazole prophylaxis in HIV patients increase the drug resistance of pneumococci? A comparative cross-sectional study in southern Ethiopia
Source: PLoS One. 2020 Dec 7;15(12):e0243054. doi: 10.1371/journal.pone.0243054 (PMC7721141; doi:10.1371/journal.pone.0243054)
Supplement: S1 File — (DOCX) [file pone.0243054.s001.docx]

# S1 File: Subject information sheet

Dear participants: good morning/good afternoon?

English Version

Title: Does cotrimoxazole prophylaxis in HIV patients increase the drug resistance of pneumococci ?. A comparative cross sectional study in southern Ethiopia

**Principal Investigator**: ……………

**Name of Organization**: Department of Medical Laboratory Sciences and Pathology, College of Health Sciences, Jimma University.

**Purpose:** The purpose of this study is to determine the impact of Cotrimoxazole Prophylaxis on Nasopharyngeal colonization and Antibiotic Susceptibility Pattern of *S. pneumoniae* among HIV Patients at ART Clinic of Arba Minch Hospital, Arba Minch, southern Ethiopia.

Cotrimoxazole prophylaxis has long been recommended for HIV-infected adults and children born to HIV infected women for prevention of *Pneumocystis jiroveci* pneumonia and toxoplasmosis, but little is known about the effects of long term use of this broad spectrum drug on the colonization rate and selection of resistance by *S. pneumoniae*.

**Duration:** The duration of this study depends upon the availability of study subjects and it might take about three months or more.

**Procedures to be carried out:** We kindly invite you to take part in this study; and this will not be successful without your participation. If you are voluntary to participate, you are expected to understand and sign the informed consent. Then, socio demographic and clinical information related to pneumococcal colonization which is important for this study will be taken from you. Nasopharyngeal swab samples will be collected by trained health officers. Collected samples will be transported to microbiology laboratory as soon as possible and will be analyzed for the presence of pneumococci by using standard operating procedures.

**Risk and discomfort:** There is almost no risk associated with the specimen collection with the exception of little discomfort because the collection of these specimens will follow the routine procedures related to laboratory investigation.

**Expected benefits:** In this study, you are not directly benefited however; the policy makers and physician will cognize the resistance patterns to prescribe an empirical antibiotic treatment.

**Confidentiality:** All your personal information collected for the purpose of this study will be kept confidential.

**Payment:** No payment will be provided for you for participating in this study.

**Right to refuse or withdraw:** Participation in the study is voluntary, and refusal to participate involves no penalty or will not compromise the health services you get at the health institutions in any way at any time. The study participants have the right to withhold information, decline to cooperate in the study and refuse provision of specimens, at any point of time.

**Approval:** This research project received ethical clearance from the ethical committee of Jimma University College of Health Sciences, Jimma University (IRB/2531/2018).

**Whom to contact:** If you have any question or need description about this study, you can communicate to the following address:

**Use the following address for any question:**

Mr. Mohammed Seid, Phone No +251-912099125, Email: m[ohammedseid2005@gmail.com](mailto:ohammedseid2005@gmail.com)
