# Supplementary material for: Does cotrimoxazole prophylaxis in HIV patients increase the drug resistance of pneumococci? A comparative cross-sectional study in southern Ethiopia
Source: PLoS One. 2020 Dec 7;15(12):e0243054. doi: 10.1371/journal.pone.0243054 (PMC7721141; doi:10.1371/journal.pone.0243054)
Supplement: S2 File — (DOCX) [file pone.0243054.s002.docx]

# S2 File: Informed consent form

Department of Medical Laboratory Sciences and Pathology, Jimma University College of Health Sciences.

Code No

Age Sex

I have been informed by Mr. Mohammed Seid, Department of Medical Laboratory Sciences and Pathology, Jimma University about a study that propose to investigate the impact of cotrimoxazole prophylaxis on non-susceptibility to antibiotics in *Streptococcus pneumoniae* isolates among HIV patients. For this study, I have been requested to participate and to give a nasopharyngeal sample. The investigator has briefed me that there are no major risks associated with the procedure. The investigator also informed me that all my response and laboratory results would be kept confidential. Moreover, I have also been well-informed of my right to withdraw from participating. I have been given enough time to think over before I signed this informed consent. It is therefore, with full understanding of the situation that I have given my informed consent and extended the cooperation at my will.

Name (participant) Signature Date

Name (data collector) Signature Date
