# Supplementary material for: Does cotrimoxazole prophylaxis in HIV patients increase the drug resistance of pneumococci? A comparative cross-sectional study in southern Ethiopia
Source: PLoS One. 2020 Dec 7;15(12):e0243054. doi: 10.1371/journal.pone.0243054 (PMC7721141; doi:10.1371/journal.pone.0243054)
Supplement: S3 File — (DOCX) [file pone.0243054.s003.docx]

# S3 File: Questionnaire

Jimma University College of Health Science, Department of Medical Laboratory Sciences and Pathology

Questionnaire to assess socio-demographic and clinical factors for nasopharyngeal colonization of drug resistance *S. pneumoniae* among HIV Patients receiving CTX and HIV-infected persons not receiving CTX and healthy controls

I am Mr. Mohammed Seid from the Department of Medical Laboratory Sciences and Pathology, Jimma University. Currently I am doing a research entitled “Does cotrimoxazole prophylaxis in HIV patients increase the drug resistance of pneumococci ?. A comparative cross sectional study in southern Ethiopia”

The objective of this questionnaire is to collect data from HIV patients and healthy controls in Arba Minch, Ethiopia, to compare pneumococcal nasopharyngeal colonization rate and drug susceptibility patterns of the isolates and to evaluate the effect of cotrimoxazole prophylaxis. It will take about 30-40 minutes to fill the questioners. You are selected to participate in this study just by chance. There are various questions that appear to be more sensitive and personal. However, we request you to give us true and right answer.

I would like to assure you the following information that you provide will be completely confidential and will be used only for the research purpose. You have the full right to refuse to take part or to stop filling the questionnaire at any time. But the information that you will provide is quite useful to achieve the objective of the study.

Your role in the success of the research is important and I appreciate your contribution to the research. Would this be ok with you?

I understood the advantage of the research and the role I am going to play in it. I have agreed to participate in the research. A. Yes B. No

Date of data collection -------------------------------------

Name of data collector -------------------------------------

Name of supervisor ------------------------------------

Hospital code__________________

|  | Questions and filters | | Coding categories | | Skip to | code | | |
| --- | --- | --- | --- | --- | --- | --- | --- | --- |
|  | Age | | --------- Years | |  |  | | |
|  | Grade level | | -----------Grade | |  |  | | |
|  | Religion | | 1.Orthodox 4. Catholic  2. Muslim 5.Other, specify-  3.Protestant | |  |  | | |
|  | Place of residence | | 1. Urban 🞎 2. Rural 🞎 | |  |  | | |
|  | Do you smoke /is there any smoker in your living house ? | | 1. Yes 🞎 2. No 🞎 | |  |  | | |
|  | How many other/family member living with you? | | 1.one-Three  2.Four and above | |  |  | | |
|  | Do you have contact with children< 5 y in the living house or work place? | | 1. Yes 🞎 2. No 🞎 | |  |  | | |
|  | | | | | | | | |
|  | Questions and filters | Coding categories | | Skip to | | | code |  |
|  | CD_4_ count | 1. -------cells/mm^3^ | |  | | |  |  |
|  | CTX prophylaxis | 1. Yes 🞎 2. No 🞎 | |  | | |  |  |
|  | If yes for Q. No.­ 9  What dose of the drug he/she is taking? | 1. Single dose 🞎 2. Double dose 🞎 | |  | | |  |  |
|  | For how long the clients have been under CTX prophylaxis | 1. Two-five month 🞎 2. Six to and above six month | |  | | |  |  |
| 12 | Did you use any other antibiotics other than CTX for the past in 2 weeks? | 1. yes 🞎 2. No 🞎 | |  | | |  |  |
| 13 | Have you started taking HAART? | 1. Yes 🞎 2. No 🞎 | |  | | |  |  |
| 14 | Do you have any history of URTI? | 1. Yes 🞎 2. No 🞎 | |  | | |  |  |
| 15 | If yes for Q. No.­ 14  Which type of URTI you suffered? | 1. Tonsillitis 🞎 2. Pharyngitis 🞎 3. Sinusitis 🞎 4. If any other specify 🞎 | |  | | |  |  |
| 16 | Do you have any LRTI? | 1. Yes 🞎 2. No 🞎 | |  | | |  |  |
| 17 | If yes for Q. No. 16  What type of LRTI you suffered? | 1. Pneumonia 🞎 2. If any other specify 🞎 | |  | | |  |  |
