# Supplementary material for: Does cotrimoxazole prophylaxis in HIV patients increase the drug resistance of pneumococci? A comparative cross-sectional study in southern Ethiopia
Source: PLoS One. 2020 Dec 7;15(12):e0243054. doi: 10.1371/journal.pone.0243054 (PMC7721141; doi:10.1371/journal.pone.0243054)
Supplement: Attachment — Submitted filename: Responses to the Reviewers.docx [file pone.0243054.s005.docx]

| **Responses to the Reviewers' comments**  Reviewers’ comments were considered and carefully revised the article as per the suggestions and comments. We are very much thankful for the Reviewers’ for their comments and suggestions which had substantially improved the quality of the manuscript. | |
| --- | --- |
| Editors comment | **Responses to the Reviewers' comments** |
| Title: remove “:-“ | Removed in the revised manuscript |
| Line 62: use “,” instead of “;” | Included in the revised manuscript |
| Line 81: write “AIDS” | Its not actually the word AIDS rather the verbal form of the word aid. And its corrected as adding in the revised manuscript |
| Table 1: maybe remove religion part from the table, data could stigmatize some group of people | Removed in the revised manuscript |
| Line 285: “common” - the 6% difference is too low for saying that the pneumococcal colonization is common in compare to control group. | Corrected as reasons” in the revised manuscript |
| Line 449-451: lack of reference | Two new references have been added in the revised manuscript. |
